# Polyvinyl chloride-added dibutyl adipate for high-performance electrohydrodynamic pumps

**DOI:** 10.3389/frobt.2023.1109563

**Published:** 2023-03-29

**Authors:** Keita Shimizu, Kazuya Murakami, Naoki Ogawa, Hideko Akai, Jun Shintake

**Affiliations:** ^1^ Department of Mechanical and Intelligent Systems Engineering, School of Informatics and Engineering, The University of Electro-Communications, Chofu, Tokyo, Japan; ^2^ Polymer Laboratory, Science and Innovation Center, Mitsubishi Chemical Co, Ltd., Yokohama-Shi, Kanagawa, Japan

**Keywords:** electrohydrodynamics (EHD), pump, dibutyl adipate, polyvinyl chloride (PVC), soft robotics

## Abstract

Electrohydrodynamic (EHD) pumps are a promising driving source for various fluid-driven systems owing to features such as simple structure and silent operation. The performance of EHD pumps depends on the properties of the working fluid, such as conductivity, viscosity, and permittivity. This implies that the tuning of these parameters in a working fluid can enhance the EHD performance. This study reports a method to modify the properties of a liquid for EHD pumps by mixing an additive. Specifically, dibutyl adipate (DBA) and polyvinyl chloride (PVC) are employed as the working fluid and the additive, respectively. The results show that when the concentration of PVC is 0.2%, the flow rate and pressure at applied voltage of 8 kV take highest value of 7.85 μL/s and 1.63 kPa, respectively. These values correspond to an improvement of 109% and 40% for the flow rate and pressure, respectively, compared to the pure DBA (PVC 0%). When the voltage is 10 kV, the flow rate of 10.95 μL/s and the pressure of 2.07 kPa are observed for DBA with PVC concentration of 0.2%. These values are more than five times higher than those observed for FC40 at the same voltage (2.02 μL/s and 0.32 kPa). The results also suggest that optimal conductivity and viscosity values exist for maximizing the EHD performance of a liquid. This demonstrates the validity of the proposed method for realizing high-performance EHD pumps by using additives in the working fluid.

## 1 Introduction

Electrohydrodynamic (EHD) pumps generate liquid flow by electrical input applied through at least one pair of electrodes (Bart et al., 1990; Chen et al., 2003; Seyed-Yagoobi, 2005; Pearson and Seyed-Yagoobi, 2009; Ramos, 2011; Fylladitakis et al., 2014; Johnson and Go, 2017). The structure of EHD pumps is simple with no moving parts assuring silent operation. The simplicity of the structure enables tailoring EHD pumps in various designs and sizes. These features make them a promising driving source for a broad range of fluid-driven systems in various fields. Particularly, in the field of soft robotics (Rus and Tolley, 2015; Polygerinos et al., 2017; Rich et al., 2018; Shintake et al., 2018), EHD pumps have attracted much attention as a key component for operating fluid-driven soft actuators (Gorissen et al., 2017; Polygerinos et al., 2017; Pagoli et al., 2022), which are the most widespread actuator type in the field. The fluid-driven soft actuators are being applied to robots of different types as well as wearable devices. For this reason, several flexible and stretchable EHD pumps have been developed (Cacucciolo et al., 2019; Mao et al., 2020; Mao et al., 2021; Seki et al., 2020; Tang et al., 2021).

An EHD pump consists of one or more pairs of positive and negative electrodes arranged in a fluid channel. By applying a voltage to the electrodes, electric charges are injected into a dielectric liquid filling the channel, which is accelerated by electric fields created between the opposing electrodes. In this phenomenon, material properties of the working fluid such as viscosity, conductivity, and permittivity determine the magnitude of flow rate and pressure (Ramos, 2011). In this context, researchers have investigated the EHD characteristics of various liquids (Crowley et al., 1990; Raghavan et al., 2009). According to the reference (Yokota, 1997), the conductivity and viscosity of liquids suitable for EHD pumps converge within a triangle on the origin side, where the viscosity and conductivity range between 1.0 × 10^−4^ Pa·s and 1.0 × 10^0^ Pa·s and between 1.0 × 10^−4^ Pa·s and 1.0 × 10^0^ Pa·s, respectively. This suggests that viscosity and conductivity are the main parameters that determine EHD characteristics and that tuning these material properties may lead to enhanced performance of EHD pumping. One way to modify the properties of a working fluid is to mix it with additives.

This study proposes and experimentally validate the use of polyvinyl chloride (PVC) as an additive to dibutyl adipate (DBA) as a working fluid for EHD pumps. PVC is a resin synthesized by the addition polymerization of vinyl chloride (CH2 = CHCl) and is widely used for insulation and piping. Adding PVC to DBA can modify the EHD characteristics and improve the resultant performance of the pump. The objective of this paper is to clarify the EHD properties of PVC-added DBA through experiments using a pump platform. It is shown that the EHD characteristics of the liquid can be tuned by the amount of the PVC additive and that the performance of the pump can be improved.

## 2 Materials and methods

### 2.1 Preparation of polyvinyl chloride-added dibutyl adipate

PVC [Shin-Etsu Chemical, TK-500 (average degree of polymerization 520)] was dissolved in DBA (Tokyo Kasei Kogyo, CAS: 105-99-7) by stirring for 30 min while maintaining the temperature at 100°C in a ceramic hot stirrer (Azwan, CHPS-170DF). Three solutions with mass concentrations of PVC 0.1%, 0.5%, and 1% were prepared. A portion of the 0.1% solution was placed in a PP container (SUNPRA-Tech, SUNPRA R-No2) and diluted two-fold with the same amount of DBA to prepare a 0.05% solution. Similarly, a 0.3% solution was prepared by diluting the 0.5% solution with DBA. All the solutions were stored at room temperature (∼23°C).

### 2.2 Fabrication of pump platform

**FIGURE 1:**
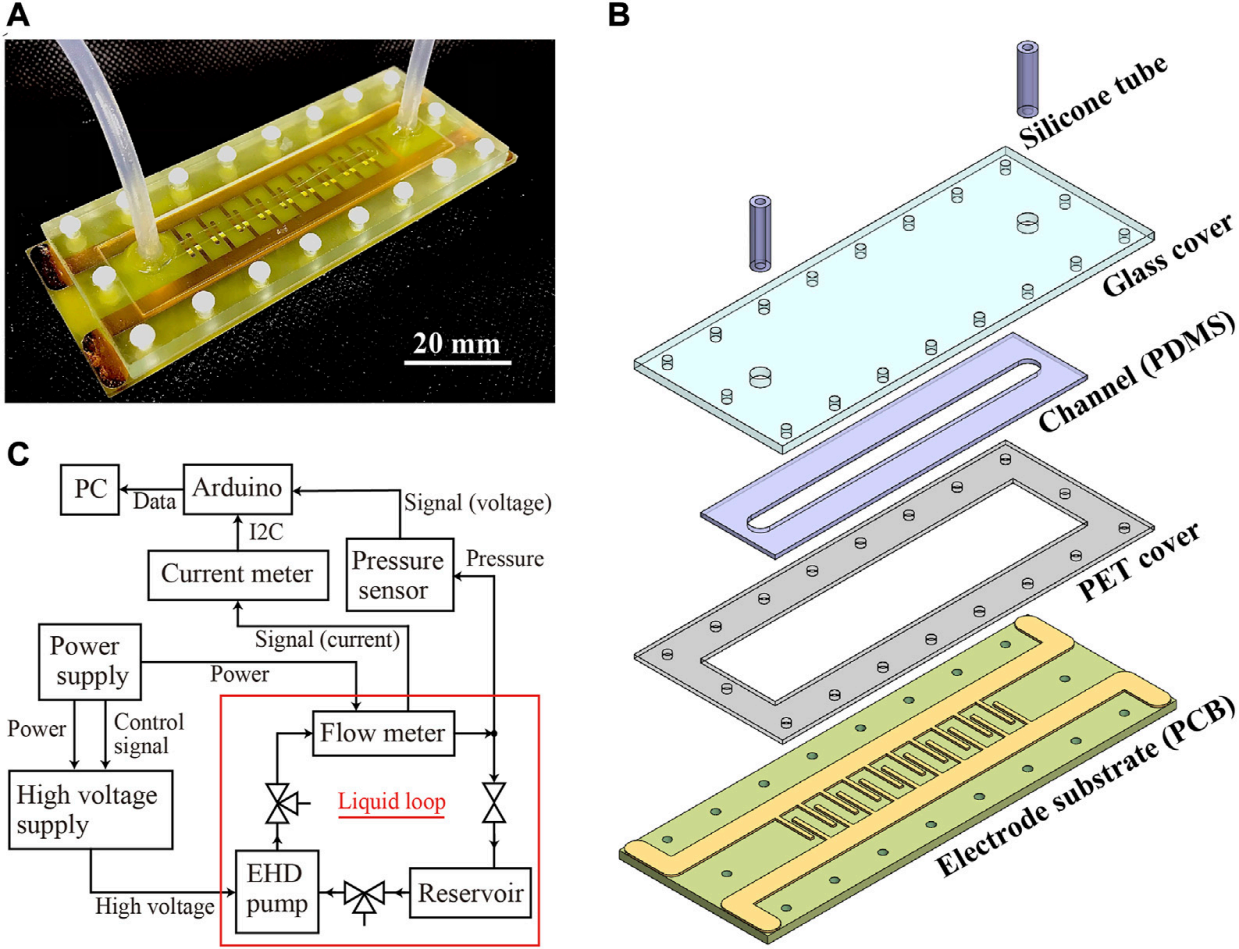
**(A)** EHD pump platform used in this study. **(B)** Structure of the pump platform. **(C)** Experimental setup used for characterizing EHD performance of tested liquids.

The pump platform used to investigate EHD characteristics of PVC-added DBA is shown in Figure 1A. It mainly consists of four components: a glass cover, a polyethylene terephthalate (PET) cover, a channel substrate and an electrode substrate, as shown in Figure 1B. Silicone tubes are connected to the channel through the upper and lower covers, allowing the input and output of a liquid. The channel is in contact with the electrode substrate (PCB), and the liquid passing through the channel is driven by applying a voltage to the electrodes.

The glass cover (thickness 5 mm) has two holes of 4 mm diameter in which a silicone tube (outer diameter: 4 mm; inner diameter: 2 mm) is placed. The channel substrate is made of 1 mm-thick polydimethylsiloxane (PDMS) and has a 5 mm-wide and 70 mm-long liquid path. The channel substrate is inserted in the PET cover (thickness 0.5 mm). The electrode substrate (PCB) has a thickness of 1.6 mm, and on top of it interdigitated electrodes are patterned, where each pair of digits has a width and gap of 1 mm each. The pairs of electrode digits are spaced 3 mm apart.

The assembly process of the pump platform was started by laying the channel substrate on top of the electrode substrate. First, a mold made of polymethyl methacrylate (PMMA) was spray-coated with a silicone release agent and then mounted on top of the electrode substrate. Next, PDMS (Dow Corning, Sylgard 184) prepared with the manufacturer recommendation ratio (raw silicone:curing agent = 10:1) was poured into the mold. The sample was placed in an oven and heated up to cure the PDMS at 80°C for 30 min. After removing the mold, the channel substrate bonded to the electrode substrate was obtained. Next, the PET cover was cut using a laser engraver (Trotec, Speedy 300) and glass the glass cover was processed by a CNC milling machine (ORIGINALMIND, KitMill CL420). These parts were stacked on the electrode substrate with a PDMS channel and fixed with plastic M2 bolts and nuts. Finally, silicone tubes were inserted and adhered using instant adhesive (Cemedine, PPX), followed by filling the holes of the upper cover with PDMS (cured at 80°C for 30 min).

### 2.3 Experimental setup

In this study, the relative permittivity, conductivity, and viscosity of the testing liquid were measured. Then, EHD pressure and flow rate generated from these liquids were acquired using the pump platform. Through these measurements, 10 liquids were investigated: pure DBA (PVC 0%), PVC-added DBA (0.05%, 0.15%, 0.2%, 0.25%, 0.3%, 0.4%, 0.5%, and 1.0% concentration), and FC40 (3M Science applied to life, 2022). FC40 is employed as a reference liquid, which is a dielectric fluid often used in EHD pumps as a working fluid.

The relative permittivity was measured using an LCR meter (NF Circuit Design Block, ZM23710) with electrode cells for liquids (Toyo Technica, LE-21). Then, 1 mL of the sample was measured at a temperature of 23°C, a voltage of 5 Vrms, an electrode spacing of 1.5mm, and a frequency of 1 kHz. The viscosity was measured at 23°C using a viscometer (BROOKFIELD, LVDV3T) with a spindle (BROOKFIELD, CPA40Z). The conductivity was measured using a digital ultra-high resistance/microcurrent meter (ADC, ADCMT5450) with electrode cells for liquids at a temperature of 23°C, a voltage of 5 Vrms, and an electrode spacing of 1.5 mm. For these tests, pure DBA (PVC 0%) and PVC-added DBA with concentration of 0.1%, 0.15%, 0.2%, 0.25%, 0.5%, and 1.0% were used.

The flow rate and pressure of liquids generated through the EHD pump platform were measured in the setup shown in Figure 1C. The setup consisted of a flowmeter (Serpas Industries, NTFZ-3S-5), pressure gauge (Panasonic, ADP5160), 3-way valve, roller clamp, reservoir tank, and pump platform. The flow paths were connected in a circulating manner, the flow rate was measured with the roller clamp open, and the pressure was measured with the roller clamp closed. The EHD pump was operated by a high-voltage power supply (EMCO, CB101), which was connected to a 4-channel power supply (Kikusui, PMX32-2QU). The values from the flowmeter and pressure gauge were recorded by a PC *via* an Arduino UNO with a sampling frequency of 10 Hz. With this setup, pure DBA (PVC 0%) and PVC-added DBA with concentration of 0.05%, 0.15%, 0.2%, 0.25%, 0.3%, 0.4%, and 0.5% were characterized at the applied voltage of 8 kV to investigate the effect of PVC addition. Then, pure DBA (PVC 0%), PVC-added DBA (0.2%), and FC40 were characterized at the applied voltage of 10 kV to compare their EHD performance. In these experiments, every liquid was measured their EHD performance three times and the average was reported.

**FIGURE 2:**
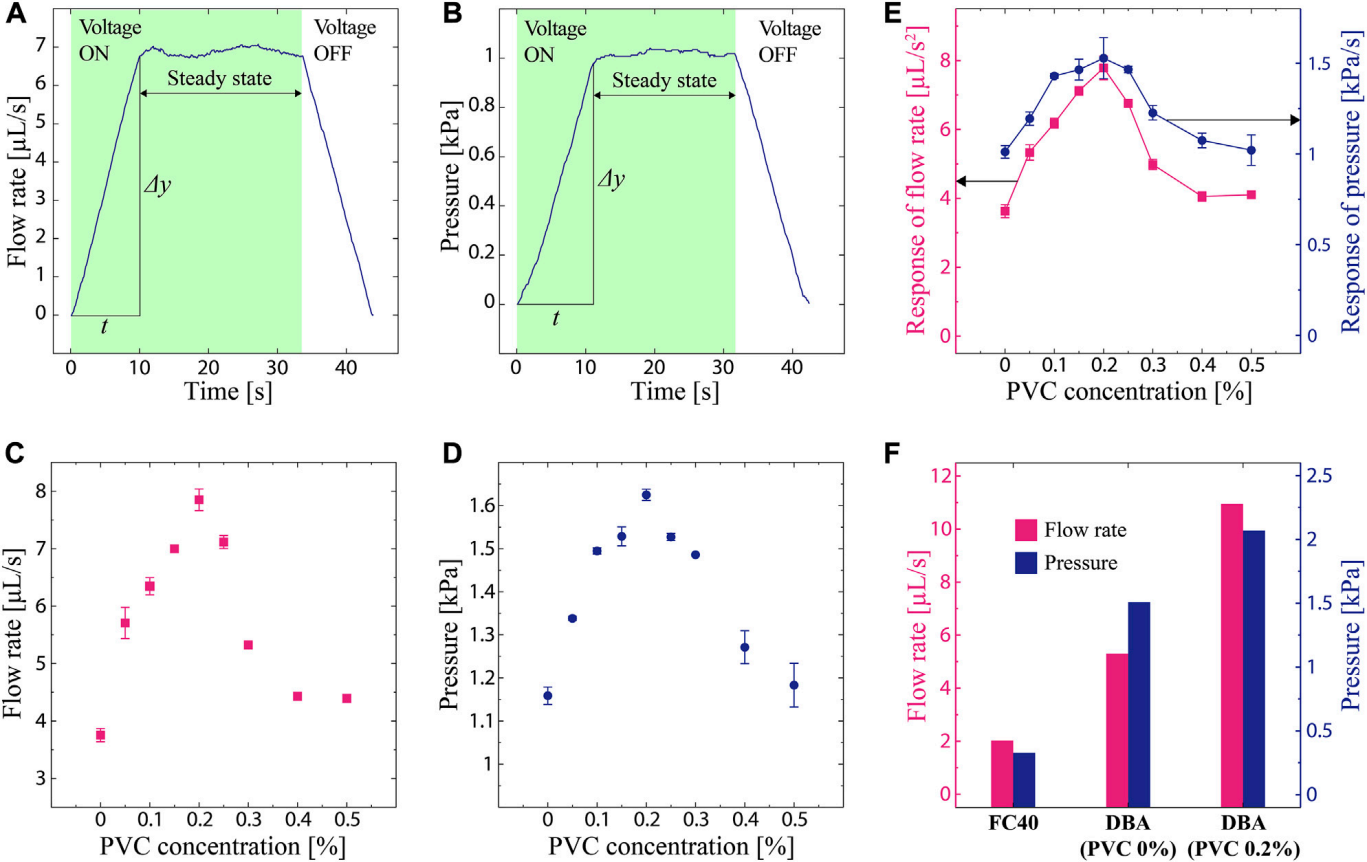
Measured EHD performance of tested liquids. **(A,B)** Typical measured data obtained through the experimental setup. **(A)** Flow rate as a function of time. **(B)** Pressure as a function of time. **(C)** Flow rate as a function of the PVC concentration (applied voltage 8 kV). **(D)** Pressure as a function of the PVC concentration (applied voltage 8 kV). **(E)** Flow rate response and pressure response as a function of the PVC concentration (applied voltage 8 kV). **(F)** Flow rate and pressure for pure DBA (PVC 0%), PVC-added DBA (0.2%), and FC40 (applied voltage 10 kV).

Figures 2A, B show the typical measured data of flow rate and pressure, respectively. The data are presented as a 100-point moving average. As indicated in the figure, the pump was activated for more than 30 s, and the average value in the steady state was considered the final measured performance. The response speed (*Δy*/*t*) of both flow rate and pressure was calculated from the amount of increase (*Δy*) immediately after the application of voltage and the elapsed time (*t*).

## 3 Result and discussion

The measured relative permittivity of pure DBA is 5.9, which is roughly three times higher than that of FC40 at 1 kHz [1.9 (3M™ Fluorinert™ Electronic Liquid FC-40 | 3M United States)]. The change in relative permittivity for PVC-added DBAs is within the range of 5.2–5.9. This suggests that DBA may enable higher EHD performance than FC40, whereas the influence of PVC addition on the relative permittivity is almost negligible. Table 1 summarizes the result.

**TABLE 1:** Measured conductivity and viscosity for the DBA with different PVC concentrations.

Liquid	Relative permittivity	Conductivity [×10^−9^ S/m]	Viscosity [×10^−3^ Pa·s]	Flow rate [µL/s]	Pressure [kPa]
DBA (PVC 0%)	5.85	2.4	5.11	3.75	1.158
DBA (PVC 0.1%)	5.26	26.1	5.30	6.35	1.494
DBA (PVC 0.15%)	5.20	52.4	5.51	7.00	1.529
DBA (PVC 0.2%)	5.23	61.0	5.67	7.85	1.625
DBA (PVC 0.25%)	5.29	88.5	5.74	7.12	1.527
DBA (PVC 0.5%)	5.25	96.2	6.68	4.39	1.183
DBA (PVC 1.0%)	5.46	103.0	9.73	N/A	N/A
FC40	1.90	0.000025	4.1	2.02	0.329

Regarding the relationship between the measured viscosity and conductivity summarized in Table 1, the viscosity increases with increasing concentration of PVC from 5.1 × 10^−3^ Pa·s to 9.7 × 10^−3^ Pa·s (PVC concentration 0%–1.0%). Similarly, the conductivity increases at higher concentrations from 2.4 × 10^−9^ S/m to 103.0 × 10^−9^ S/m (PVC concentration 0%–1.0%). These results indicate that the viscosity and conductivity can be tuned by adjusting the amount of PVC addition. However, it should be noted that these two parameters are mutually dependent and their change against the amount of PVC is sensitive. Therefore, careful tuning is required to achieve optimal characteristics.

The measured flow rate as a function of PVC is plotted in Figure 2C. Pure DBA exhibited a flow rate of 3.75 μL/s. When PVC is added to DBA, the flow rate increases and achieves a peak value of 7.85 μL/s at 0.2% concentration, which is a 109% improvement compared to pure DBA. Above the concentration of 0.2%, the flow rate decreased as the concentration reached 0.5%.

The measured pressure shows a similar trend as that shown in Figure 2D. Pure DBA generates a pressure of 1.16 kPa. The highest value is 1.63 kPa at 0.2% PVC concentration, which is a 40% increase compared to the pure one. These results indicate that the addition of PVC to DBA improves the output performance of EHD, which has an optimal PVC concentration to maximize performance.

The response rate of the flow rate and pressure shown in Figure 2E has a similar trend to the results discussed above; the performance increases when the concentration of PVC is around 0.2%. Specifically, the response of flow rate for pure DBA (3.62 μL/s^2^) gets 115% improvement (7.79 μL/s^2^) at 0.2% concentration of PVC addition. As for the response of pressure for pure DBA, it exhibits 1.01 kPa/s. The pressure response is the highest at 0.2% PVC concentration and shows a value of 1.53 kPa/s, corresponding to a 51% increase compared to pure DBA. The data suggest that adding PVC into DBA improves not only the magnitude of EHD output but also the response or the time at which the output reaches the steady state. Moreover, the response speed of both flow rate and pressure is maximized at a certain PVC concentration.

Figure 2F plot the measured flow rate and pressure for pure DBA (PVC 0%), PVC-added DBA (0.2%), and FC40 tested at voltage of 10 kV. PVC-added DBA (0.2%) exhibits flow rate and pressure as 10.95 μL/s and 2.01 kPa, which are more than five times higher than those of FC40 (2.02 μL/s and 0.33 kPa).

**FIGURE 3:**
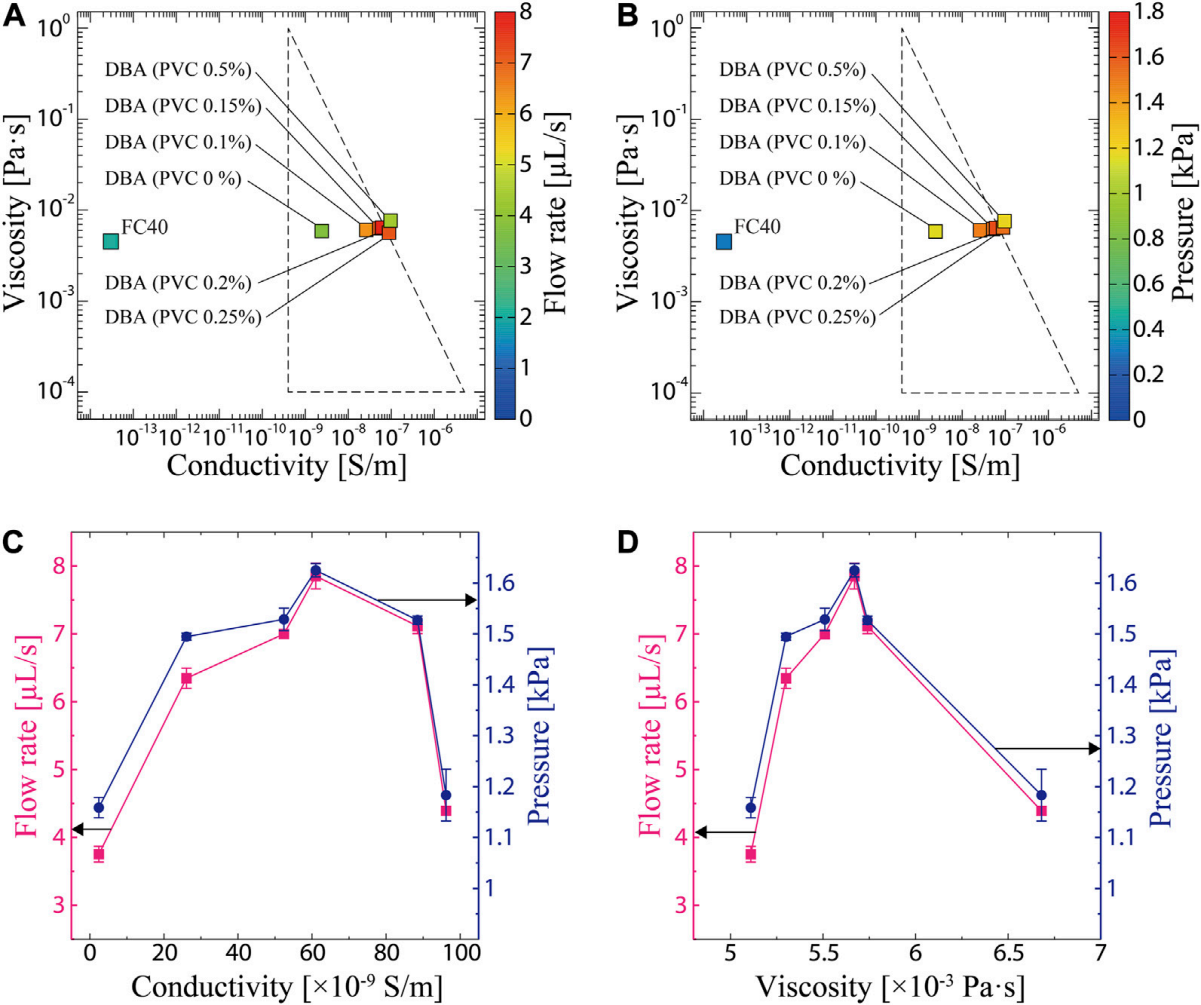
**(A)** Measured viscosity, conductivity, and flow rate of tested liquids. **(B)** Measured viscosity, conductivity, and pressure of tested liquids. **(C)** Relationship between the conductivity, flow rate, and pressure. **(D)** Relationship between the viscosity, flow rate, and pressure.

Figures 3A, B map the conductivity and viscosity of the liquids tested in this study and present the measured flow rate and pressure using a color bar. The triangle in each figure represents the range of viscosity and conductivity suitable for working fluids of EHD reported in the literature (Yokota, 1997). The conductivity and viscosity of pure DBA and PVC-added DBA match the triangular range, confirming their suitability as working fluids. FC40 and DBA with PVC concentration of higher than 0.2% are outside the range, which is in agreement with the experimental results, that is, these liquids generate lower performance compared to the those with PVC concentration of 0%–0.2%. To further clarify the relationship between the material properties and the EHD performance, the flow rate and pressure in the triangle range as a function of the conductivity and viscosity, respectively, have been plotted in Figures 3C, D. The flow rate and pressure rise and fall as the conductivity increases with PVC addition to DBA, achieving a peak at 61.0 × 10^−9^ S/m. The same trend appears when the PVC concentration in DBA is increased, where both the flow rate and pressure exhibit a peak value at 5.7 × 10^−3^ Pa·s. These results indicate that there exist optimal values of conductivity and viscosity that maximize the EHD performance. It should be noted that, in general, low viscosity is preferred for working fluids of EHD as it leads to smooth flow. Hence, it is assumed that the main material property contributing to the improvement of the EHD performance may be conductivity. This also implies that higher performance may be achieved when the viscosity is lowered while maintaining optimal conductivity.

## 4 Concluding remarks

In this study, adding PVC into DBA is proposed to produce working fluids that enable the high performance of EHD pumps. To validate the effectiveness of the method, DBA with different PVC concentrations and FC40 are tested in a pump platform, in which the EHD performance is measured in terms of the flow rate and pressure. FC40, which is often used in EHD pumps, is employed as the reference liquid. The experimental data shows that the addition of PVC into DBA improves the flow rate and pressure by 109% and 40%, respectively. This improvement is achieved at PVC concentration of 0.2%, which provides EHD performance of more than five times higher than FC40. The data also suggest the presence of optimal conductivity and viscosity values that maximize the EHD performance. These results validate the method proposed in this study—the use of additives for working fluids enabling enhanced EHD performance.

Future work involves the investigation of EHD characteristics for different combinations of liquids and additives. In this work, the relationship between the conductivity and viscosity of liquids is further clarified. These studies can lead to the realization of high-performance EHD pumps, which paves the way for advanced fluid-driven systems including soft robots and wearable devices.

## Data Availability

The raw data supporting the conclusion of this article will be made available by the authors, without undue reservation.
